# The Senses of Agency and Ownership in Patients With Borderline Personality Disorder

**DOI:** 10.3389/fpsyt.2020.00474

**Published:** 2020-06-09

**Authors:** Tim Julian Möller, Niclas Braun, Ann-Kathrin Thöne, Christoph S. Herrmann, Alexandra Philipsen

**Affiliations:** ^1^ Berlin School of Mind and Brain, Humboldt University of Berlin, Berlin, Germany; ^2^ Experimental Psychology Lab, Department for Psychology, Faculty for Medicine and Health Sciences, Carl von Ossietzky University, Oldenburg, Germany; ^3^ Department of Psychiatry and Psychotherapy, University of Bonn, Bonn, Germany; ^4^ Department of Psychiatry, Faculty for Medicine & Health Sciences, University of Oldenburg, Oldenburg, Germany; ^5^ School of Child and Adolescent Cognitive Behavior Therapy (AKiP), Faculty of Medicine and University Hospital Cologne, University of Cologne, Cologne, Germany

**Keywords:** borderline personality disorder, sense of ownership, sense of agency, sense of self, active rubber hand illusion, consciousness, bodily awareness

## Abstract

Patients with borderline personality disorder (BPD) not only experience a strong instability in their affect and interpersonal relations but also disturbances in their self-experience, including dissociation and body-alienation symptoms. It is not yet understood whether an altered sense of ownership (SoO) or sense of agency (SoA) may contribute to these disturbances. One recent hypothesis is that patients with BPD have a reduced sense of self and are therefore more likely to misattribute external objects or actions to their own self than healthy individuals. The present study followed up this hypothesis by investigating whether BPD patients have a more flexible body representation than healthy participants. More specifically, the active rubber hand illusion (aRHI) was applied to 21 patients with BPD and the same number of healthy participants. Using established subjective, electrodermal, and behavioral measures, the participants’ SoO and SoA were assessed during the aRHI. The findings show self-reported evidence for higher SoO under anatomical hand congruency as compared to anatomical incongruency, but no evidence for group differences between BPD patients and healthy participants. This finding is inconsistent with previous findings of an enhanced SoO-related body plasticity in BPD patients. Regarding SoA, the findings show self-report evidence of higher SoA in BPD patients versus healthy participants, although this group difference was not evident in the implicit SoA measure (intentional binding). In summary, the present study only reveals partial evidence for a higher body plasticity in BPD patients. Instead, the observed variability in results appears better explainable by some generally elevated perceptual suggestibility of BPD individuals.

## Introduction

Borderline personality disorder (BPD) is a mental disorder marked by a pervasively unstable pattern in affective experience, interpersonal relationships, impulsive behavior, and self-image (1). Typical clinical symptoms include affective dysregulation, impulsive aggression, non-suicidal self-injury, and suicidal behavior (2). Besides these core symptoms, patients with BPD also often demonstrate disturbances in their bodily self-experience. These disturbances not only relate to a negative bodily self-image such as an increased body dissatisfaction and lowered self-appraised body attractiveness (3, 4), but also to more global body-perceptual distortions, such as dissociation phenomena or body-alienation symptoms, including experiences of disownership toward one’s own body (5, 6).

The sense of ownership (SoO) is described as the experience of “mineness” toward one’s body, and the sense of agency (SoA) relates to the experience of being the initiator and author of an action [for a review, see Braun et al. (7)]. It is not yet understood whether an altered SoO or SoA may contribute to the distorted bodily self-experiences in BPD patients. One current hypothesis is that, similar to patients suffering from schizophrenia (8–10), patients with BPD also have a weakened sense of self that manifests itself in a disturbed SoO experience (11) as well as hyperplastic body representation (12, 13).

With regard to a disturbed SoO experience in BPD patients, Löffler et al. (11), for instance, compared the levels of SoO for 25 separate body parts between current BPD patients, remitted BPD patients, and healthy participants. One interesting outcome was that the current BPD patients reported significantly reduced whole-body SoO as compared to the healthy participants, while no significant differences were found between the current and remitted BPD patients, nor between the remitted BPD patients and healthy participants. Remarkably, reduced SoO was unequally distributed over the whole body. For instance, for the hip/buttocks, the breasts, and the genitals, lower SoO was reported than for other body parts. Moreover, for the current BPD patients, an inverse correlation was found between whole-body SoO and trait dissociation. Hence, it appears that BPD patients not only present higher levels of body dissatisfaction (3) but also tend to experientially dissociate body parts from their self.

Evidence for a hyperplastic body representation, in turn, comes from two recent body plasticity studies (12, 13) in which the rubber hand illusion (RHI) (14) was applied to BPD patients. In this paradigm, an artificial hand is placed visibly in front of the participant, while the participant’s own hand is masked. Typically, the artificial hand is placed in an anatomically plausible (i.e., congruent) position, but this position can also be experimentally manipulated. If the artificial hand and the participant’s own hand are stroked synchronously with a brush, a multimodal conflict is induced. While the stroking is observed on the artificial hand, it is felt on the real (hidden) hand. To resolve this multimodal conflict, the brain concludes that only the artificial hand exists and shifts its SoO attribution toward this hand (7). Applying the RHI paradigm to BPD patients, Bekrater-Bodmann et al. (12) found that patients with current BPD reported higher SoO toward the presented artificial hand compared to healthy participants, while no significant differences were found between patients with remitted BPD and healthy participants, nor between current and remitted BPD patients. In accordance with these findings, also Neustadter et al. (13) found that patients with BPD report higher SoO for the RHI than healthy participants. Hence, these results indicate that patients with BPD show an enhanced body plasticity, in that they appear more prone to experience non-body objects like an artificial hand as part of their own body than healthy persons.

While Löffler et al.’s (11) findings of reduced SoO toward the own body as well as Bekrater-Bodmann et al.’s (12) and Neustadter et al.’s (13) findings of enhanced SoO toward an artificial hand may appear contradictory at first sight, both findings could result from a loosened bodily boundary and less fine-grained body representation in BPD patients. That is, BPD patients might be less certain about which physical objects form part of the own body, and which do not. As has been indicated by RHI studies in other populations, it appears that the more impaired the own body representation, for instance, due to hemiplegia (15) or mechanical limb immobilization (16), the higher is typically the susceptibility toward the RHI. Respectively, the more fine-grained the own body representation, for instance, in expert pianists (17), the lower the susceptibility toward the RHI.

While Bekrater-Bodmann et al.’s (12) and Neustadter et al.’s (13) RHI studies provided valuable insights into altered body plasticity in BPD patients, both studies also had some limitations: First, a stronger level of embodiment was only found for the SoO questionnaire data, while in neither of these studies, significant group differences could be deduced from the proprioceptive drift measure (i.e., an implicit SoO measure where the participants have to blindly indicate the felt location of their real hand, which they typically mislocate toward the artificial hand). Since patients with BPD tend to have an especially high response bias (18), one should, however, be cautious to interpret self-report data in isolation. Second, both studies only recruited female participants, which limits extrapolating these results to the general BPD population. Third, both studies did not investigate the role of SoA in BPD. As has been indicated by several SoO/SoA combination studies (19–27), there is a complex interplay between SoO and SoA in healthy participants [for a review, see Braun et al. (7)]. While, for instance, controversy exists in respect to whether SoO is promoted by voluntary action (SoA) (19, 20, 23), or not (27), the overall pattern indicates that although both experiences can be experimentally dissociated to some extent (19, 20), they typically strengthen each other (19, 20, 23). Hence, the question arises whether SoA is also affected in patients with BPD, and if so, how it relates to SoO in this population.

For these reasons, further research is necessary to specifically investigate SoO and SoA in BPD using behavioral and physiological measures. An improved understanding of the distinct mechanisms might also contribute to more advanced techniques to operationalize measurements and to ultimately unravel the mechanisms behind self-disturbances.

Therefore, the present study investigates SoO and SoA and their complex interplay in patients with BPD and healthy participants by applying the active RHI (aRHI) paradigm (19, 20). In contrast to the classical RHI paradigm, the aRHI is induced by synchronous movements of the artificial hand and participant’s real hand, rather than by visuotactile matching alone (7). By applying the aRHI to BPD patients, we complement Bekrater-Bodmann et al.’s (12) and Neustadter et al.’s (13) studies and respond to the above limitations. More specifically, we address three research questions: First, we investigate whether the finding of higher self-reported SoO in patients with BPD (12, 13) can be replicated. And if so, we further study whether this embodiment effect can also be physiologically demonstrated by an implicit electrodermal activity (EDA) measure (28). Second, using explicit and implicit SoA measures, we explore whether, besides SoO, SoA is also altered in patients with BPD. And finally, we investigate whether our different explicit and implicit measures of SoO and SoA converge in patients with BPD and in healthy participants.

## Materials and Methods

### Participants

Twenty-one patients with BPD (18 female) aged between 18 and 52 years, and 21 healthy participants were recruited for the study (see **Table 1** for demographic and clinical data). All patients were inpatients of the BPD special ward from the Oldenburg University Clinic for Psychiatry and Psychotherapy. For each patient with BPD, a gender-, education-, and age-matched ( ± 4 years) healthy participant was recruited *via* local announcements and bulletin boards. Handedness was assessed by self-report. All participants provided written informed consent for the study. The experiment was approved by the local medical ethics committee of the University of Oldenburg and registered at DRKS.de (DRKS00012893).

**Table 1:** Demographic and questionnaire data of BPD patients and healthy participants.

Characteristics	BPD patients (n = 21)	Healthy participants (n = 21)	
Female: n (%)	17 (80.95%)	17 (80.95%)	
Age: mean (SD)	28.4 (8.87)	30.3 (9.96)	
Handedness (left: right)	3: 18	1: 20	
Comorbid ADHD	1	0	
Comorbid mood disorder	6	0	
Comorbid panic disorder	1	0	
Questionnaire data	BPD patients (n = 20)*	Healthy participants (n = 21)	t-test
BSL-23 Score (SD)	1.40 (0.87)	0.23 (0.22)	t = 5.28; p < .001
Global Well-being Score (SD)	57.75 (14.74)	77.57 (14.40)	t = −4.04 p < .001
CDS-Score (SD)	2.00 (1.33)	0.50 (0.35)	t = 4.72; p < .001

ADHD, attention-deficit/hyperactivity disorder; BSL-23, Borderline Symptom Checklist 23; CDS, Cambridge Depersonalization Scale.

*Due to missing data, the analysis of questionnaire data is based on only 20 participants.

The final analysis of the behavioral and self-report data included 20 BPD patients and 21 healthy participants, since one BPD patient refused to fill in the questionnaires. The EDA analysis included 20 BPD patients and 19 healthy participants, since one BPD patient and two healthy participants had to be excluded from the statistical analysis due to technical malfunction.

### Patient Assessment

The BPD diagnosis followed criteria based on the Diagnostic and Statistical Manual for Mental Disorders IV (DSM-IV) (29) and was based on a 1-h semi-structured clinical interview with a psychologist that each patient of the BPD special ward went through upon admission. The BPD diagnosis was confirmed by administering the German versions of the “Borderline Symptom Checklist 23” (BSL-23) (30) and the “Assessment of DSM-IV Personality Disorders” inventory (ADP-IV) (31). The BSL-23 assesses the severity of BPD symptoms on six dimensions: “self-perception”, “dysphoria”, “loneliness”, “intrusions”, “affect-regulation”, and “self-destruction”. Moreover, it includes a global well-being scale ranging from 0 (very bad) to 100 (excellent). The ADP-IV is a 94-item questionnaire that allows for a categorical and dimensional assessment of personality disorders according to DSM-IV criteria. In addition, the German version of the “Structured Clinical Interview for DSM-IV” (SCID-I) (32) was conducted with all patients. Only patients who did not meet criteria for an Axis-I disorder were kept in the study, except for Axis-I disorders of past severe depression, current mild depression, panic disorder, or attention-deficit/hyperactivity disorder (ADHD). These inclusion criteria were formed due to the high comorbidity rates of these disorders. Finally, the 30-item “Cambridge Depersonalization Scale” (CDS-30) (33), which targets the frequency and duration of depersonalization and derealization symptoms over the last 6 months, was filled in by all BPD patients.

### Healthy Control Participant Assessment

All healthy participants were required to be free from any current Axis I or II disorders and underwent the SCID-I and ADP-IV diagnostic prior to the experiment. Only participants who did not meet diagnostic criteria for any Axis I or II disorder remained in the study.

### Apparatus and Experimental Conditions

**Figure 1:**
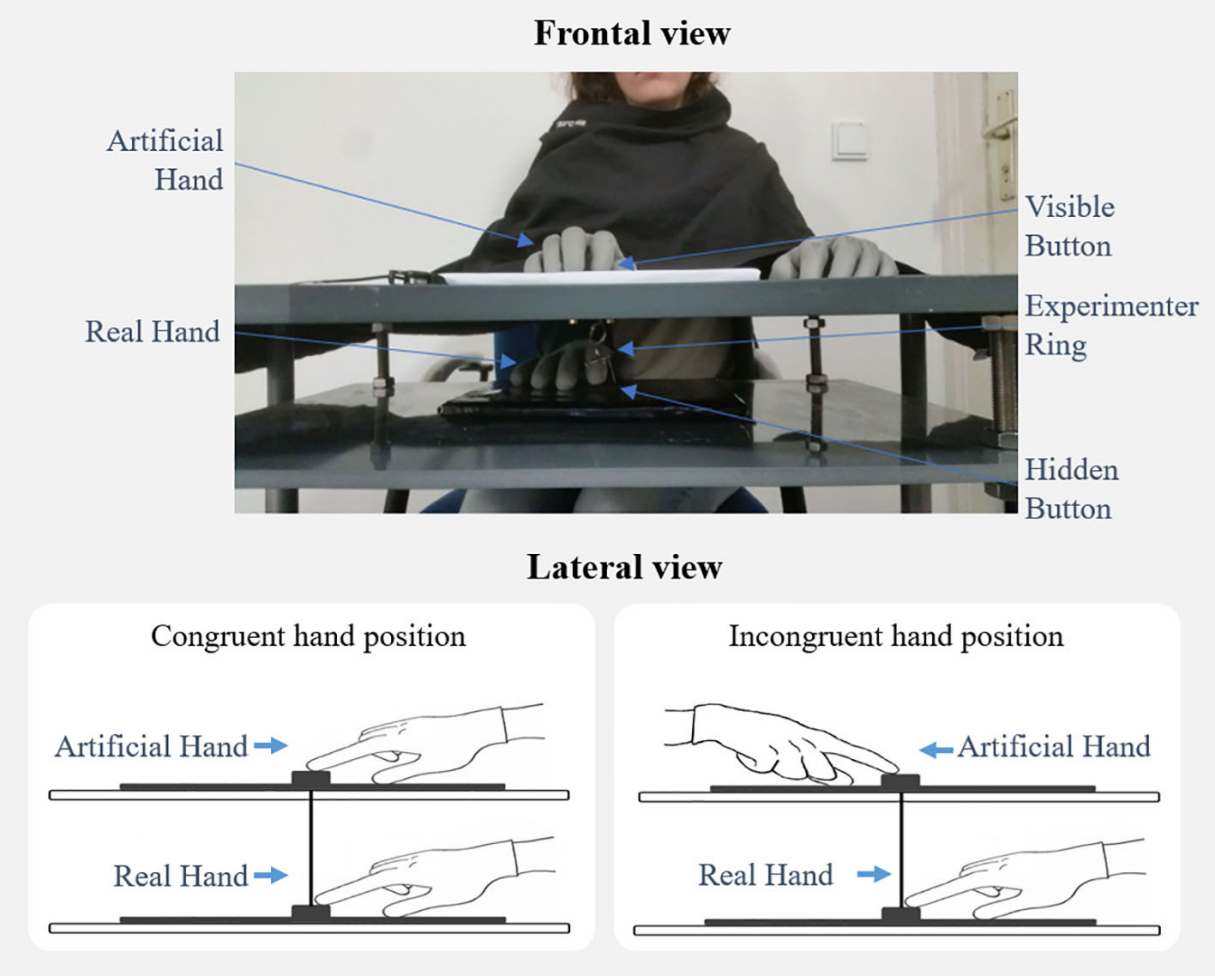
The active rubber hand illusion. The participant’s real right index finger (lower plate) is connected with the movable artificial index finger (upper plate) *via* a small string. As a result, whenever the participant moves his or her own index finger up or down (resp., presses the button), the artificial index finger moves correspondingly. If the artificial hand is thereby positioned in an anatomically plausible position (i.e., congruent hand position), this setting induces Sense of Ownership and Sense of Agency toward the artificial hand in many participants.

The aRHI apparatus used in the present experiment (**Figure 1**) was very similar to the one used in the authors’ previous aRHI study (19). The participants sat in front of a rectangular table that consisted of a tabletop and a lower plate, such that the participants’ real right hand could be placed on the lower platform and an artificial right hand on the tabletop. While in the former aRHI study (19), the vertical distance between both plates amounted to 7.5 cm, in the present study this distance amounted to 9.5 cm due to a new table with a thicker tabletop. In the middle of each platform, a button was inserted. The upper button was connected to a computer to record keypresses, using Presentation software (version 19.0; Neurobehavioral Systems Inc., Albany, USA). A plaster cast of a medium-sized artificial hand (18 cm in length, from the tip of the middle finger to the end of the wrist) was used for inducing the aRHI. Throughout the experiment, the participants placed their real right hand’s index finger on the lower button, while the artificial index finger rested on the upper button. To induce the visual impression that the artificial hand could be the participants’ own hand, both the artificial and real hands were covered with an identically looking thin-gauge garden glove. Also, an apron was placed over the participants’ shoulders and arm to cover the space between the participants’ body and artificial hand (**Figure 1**). The artificial index finger was equipped with a rebounding joint, such that it could be moved up and down. Moreover, a small string was attached to the lower side of the artificial finger’s tip and was connected through a small hole in the tabletop to the area below where it was split and attached to the outer edges of the lower button (without touching the participant’s finger). Using this setting, natural button press movements could be realistically mimicked by the artificial hand. That is, whenever the participants moved their right finger up or down, the artificial index finger and upper button moved accordingly and without any temporal delay (**Figure 1**).

Prior to the experiment, a practice run and an intentional binding control condition without the aRHI apparatus was carried out (for details, see *Intentional Binding*). In the practice run, participants repeatedly estimated the time interval between two subsequently played sounds and received feedback on their accuracy by the experimenter. In the control condition, participants again estimated the time interval between two sounds but without receiving any feedback. This condition will be hereinafter referred to as the no-agency control condition.

For the actual aRHI experiment, two conditions were employed: A spatially congruent condition, in which the artificial hand was aligned with the participant’s own hand, and a spatially incongruent condition, in which the artificial hand was rotated by 180° (i.e., placed in misalignment to the participant’s real hand). The order of the conditions was counterbalanced. That is, half of the participants started in the congruent condition and the other half in the incongruent condition. In brief, four experimental conditions were employed: 1) congruent condition in BPD patients; 2) incongruent condition in BPD patients; 3) congruent condition in healthy participants; 4) incongruent condition in healthy participants.

The experimental procedure with the aRHI apparatus was identical for both participant groups and consisted of two blocks, one for the congruent condition, and one for the incongruent condition. Both blocks were identically structured: First, an intentional binding phase took place, immediately followed by a free pressing phase, and finally, a syringe was pricked into the artificial hand. During the intentional binding and free pressing phases, participants were instructed to focus on the artificial hand and its button presses. Also, a break of approximately 3 min was included between blocks to give instructions for the next upcoming block and to rotate the table by 180°. The overall procedure took approximately 60 min and EDA was recorded throughout the experiment.

### Intentional Binding

As in previous studies (19, 34–36), SoA was implicitly assessed by the intentional binding paradigm. In the current variant of this paradigm, participants were asked to repeatedly judge the time interval between a button press and a subsequently played sound. A typical observation for this variant is that time intervals are underestimated when the button presses are voluntarily self-generated compared to when they are passively induced [e.g., by Transcranial Magnetic Stimulation (TMS) triggers] or only observed (37). In the present study, the intentional binding paradigm was directly incorporated into the aRHI, such that button presses conducted with the lower button were observed as button presses carried out by the artificial hand. For each condition, participants carried out 30 time-interval estimation trials. Each trial thereby consisted of a voluntarily button press, a subsequently played sound, and an ensuing time interval estimation. The time interval estimations were thereby verbally given to the experimenter. Following our previous study (19), 1.8 kHz sounds with a duration of 100 ms were presented *via* a notebook either 100, 400, or 700 ms after each button press onset. The latency for each sound was pseudo-randomized, such that each latency type occurred exactly 10 times. Participants, in turn, were told that the sounds were played randomly between the range of 0 ms (immediately after the button press) and a maximum delay of 1,000 ms.

Prior to the experiment, a practice run was carried out, consisting of 10 trials with the same sound latencies as in the main experiment, but without the aRHI apparatus. In this setting, the participants’ task was to repeatedly estimate the time interval between two subsequently played sounds. The purpose of this practice run was to acquaint the participants with the estimation of small time intervals, in order to minimize time misestimation effects unrelated to intentional or causal binding. After each practice trial, verbal feedback was given on the precision of the time interval estimation. However, to preserve the illusion that the second sound followed the first sound with a random delay (and not exactly after 100, 400, or 700 ms), the experimenter did not report the exact time interval, but added a randomly generated value between 1 and 99 ms to each verbal feedback. Further, the no-agency control condition was employed after the practice run (19). This condition was introduced to identify potential systematic effects of time misestimating unrelated to the intentional binding effect itself. The no-agency control condition was identical to the practice run except that no feedback on the estimation accuracy was given, and that 30 trials as for the intentional binding trials during the four experimental conditions (congruent condition in BPD patients; incongruent condition in BPD patients; congruent condition in healthy participants; incongruent condition in healthy participants) instead of only 10 trials were presented. For the statistical analysis, intentional binding was defined as the average error in the estimation (Ev) of the actual time interval (Av) in milliseconds.

$$\mathrm{IB}=\sum_{i=1}^{n_{\mathrm{Trials}}}\frac{{\mathrm{Av}}_{i}-{\mathrm{Ev}}_{i}}{n_{\mathrm{Trials}}}$$

### Free Pressing Phase and Syringe Application

The “free pressing phase” of the aRHI experiment was adapted from earlier studies (19, 20). Here, participants were asked to look at the artificial hand and to repeatedly press the button in a 1-Hz rhythm until the experimenter instructed them to stop. The regularity of the 1 Hz rhythm was not further considered and was solely used to drag the focus of the participant on the artificial hand in order to standardize, maximize, and maintain both, the SoO and SoA illusion over the artificial hand. After 1 min, the experimenter said “stop” and immediately stabbed a syringe into the rubber hand (between the index and long finger), to evaluate whether such threatening of the artificial hand leads to a transient electrodermal response.

### Stress-Level Questionnaire

A single-item questionnaire was handed to the participants immediately after syringe application. Participants were asked to rate their stress level on a Likert-scale ranging from 1 (“low”) to 7 (“high”).

### SoO and SoA Questionnaire

The 12-item SoO and SoA questionnaire (see **Table 2**) used in the former aRHI experiment (19) was also applied in the present study and was applied after each experimental block. This questionnaire is an adapted version from existing RHI questionnaires (14, 20, 38). Four statements referred to SoO (e.g., “I felt as if I was looking at my own hand”) and four to SoA (e.g.,”I felt as I were controlling the movements of the artificial hand”). The remaining four statements served as control statements and shared several similarities with illusion-specific qualities but lacked the specific phenomenal experience of ownership or agency. For example, a SoO-control question was “It appeared as if the artificial hand were drifting toward my real hand” and a SoA-control question was “It seemed as if the rubber hand had a will on its own”. For each statement, participants reported their level of agreement on a 7-point Likert scale ranging from −3 (“totally disagree”) to 3 (“totally agree”), whereby 0 indicated neither agreement nor disagreement (“neutral”). In line with previous studies (19, 20, 28, 39–43), the illusion criterion for a successful SoO and SoA induction was set to an average value of 1 (“rather agree”).

**Table 2:** Questionnaire for sense of ownership (SoO) and sense of agency (SoA) evaluation.

Phenomenal Target Property	Statement
Sense of ownership	I felt like I was looking at my own hand.
	I felt like the artificial hand was part of my body.
	It seemed as if I were sensing the movement of my finger in the location where the artificial finger moved.
	I felt as if the rubber hand were my hand.
Sense of ownership (control questions)	It appeared as if the artificial hand were drifting toward my real hand.
	It felt as if I no longer had a right hand, as if my right hand disappeared.
Sense of agency	The artificial hand moved just like I wanted it to, as if it were obeying my will.
	Whenever I moved my finger, I expected the artificial finger to move in the same way.
	I felt as if I were causing the movement that I saw.
	I felt as if I were controlling the movements of the artificial hand.
Sense of agency (control questions)	I felt as if the artificial hand were controlling my will.
	It seemed as if the rubber hand had a will on its own.

Four questions addressed each phenomenal target property. In addition, control questions were included that included illusion-related questions but did not capture specific phenomenology of SoO and SoA. The questions were read in counterbalanced order to the participants, and the participants rated their level of agreement on a 7-point Likert scale.

### EDA Recording

In line with former RHI studies, EDA was used as an implicit measure for artificial hand embodiment (28, 44–46) and analyzed for the time intervals of the syringe applications. We expected that if the artificial hand was experienced as part of the own body, this should lead to a stronger fear response, and therefore to a higher phasic EDA response, than if the artificial hand was just perceived as an external object. EDA was acquired using a BrainAmp ExG amplifier (BrainProducts GmbH, Gilching, Germany) according to previously established criteria (47). Two sintered silver/silver chloride (Ag/AgCl) electrodes were filled with a sodium chloride (NaCl) paste and were attached to the annular and middle finger of the left hand. The continuous EDA signal was filtered analogously from 0 to 1,000 Hz and digitized with a sampling frequency of 150 Hz (0.006 μS resolution) using the Brain Vision Recorder software (BrainProducts GmbH, Gilching, Germany). EDA data were analyzed with the software LEDALAB v3.4.350,51. This Matlab toolbox allows to conduct a continuous decomposition analysis (CDA) by which the EDA signal may be decomposed into its tonic and phasic EDA parts. The resulting phasic EDA part was retained and segmented from −3 to +15 s relative to the start of the application of the syringe. Baseline correction of all segments was employed by calculating percent amplitude changes, relative to the average EDA of the first 2 s time interval. For statistical analyses, the mean phasic EDA response (in %) was extracted for the +7 to +13 s interval, relative to syringe application.

### Statistical Analysis

Four experimental dependent variables were in the focus of this experiment: reported SoO, reported SoA, phasic EDA-responses, and intentional binding. These variables were monitored by three control variables: SoO control questions, SoA control questions, and the no-agency control condition of the intentional binding.

A 2 × 2 mixed factorial design with one between-subjects factor Group (BPD patients vs. healthy participants) and one within-subjects factor Position (congruent vs. incongruent) was used for each experimental dependent variable. While the Group factor distinguished between the BPD group and the group of healthy participants, the Position factor specified whether the artificial hand was placed in anatomical alignment (congruent condition) or in anatomical misalignment with the participants’ real hand (incongruent condition). For each experimental dependent variable, a separate mixed two-way ANOVA was conducted.

In addition, pairwise comparisons by means of multiple one-tailed t-tests of the perceived SoA and SoO levels with the control questions were carried out in both groups to test for a response bias and to ensure that the experimental manipulations affected only the core phenomenology at the focus of the study.

Moreover, to confirm that less temporal binding occurred in the absence of agency, multiple one-tailed t-tests between the congruent condition and the no-agency control condition, as well as between the incongruent condition and no-agency control condition were conducted separately for both groups.

Furthermore, Pearson correlation coefficients were calculated to explore the relationship for each possible combination of the experimental dependent variables. Correlation coefficients were individually computed for each condition as well as pooled across conditions.

## Results

Data were analyzed for the four experimental conditions: congruent condition in BPD patients, incongruent condition in BPD patients, congruent condition in healthy participants, and incongruent condition in healthy participants.

### Self-Report Data

**Figure 2:**
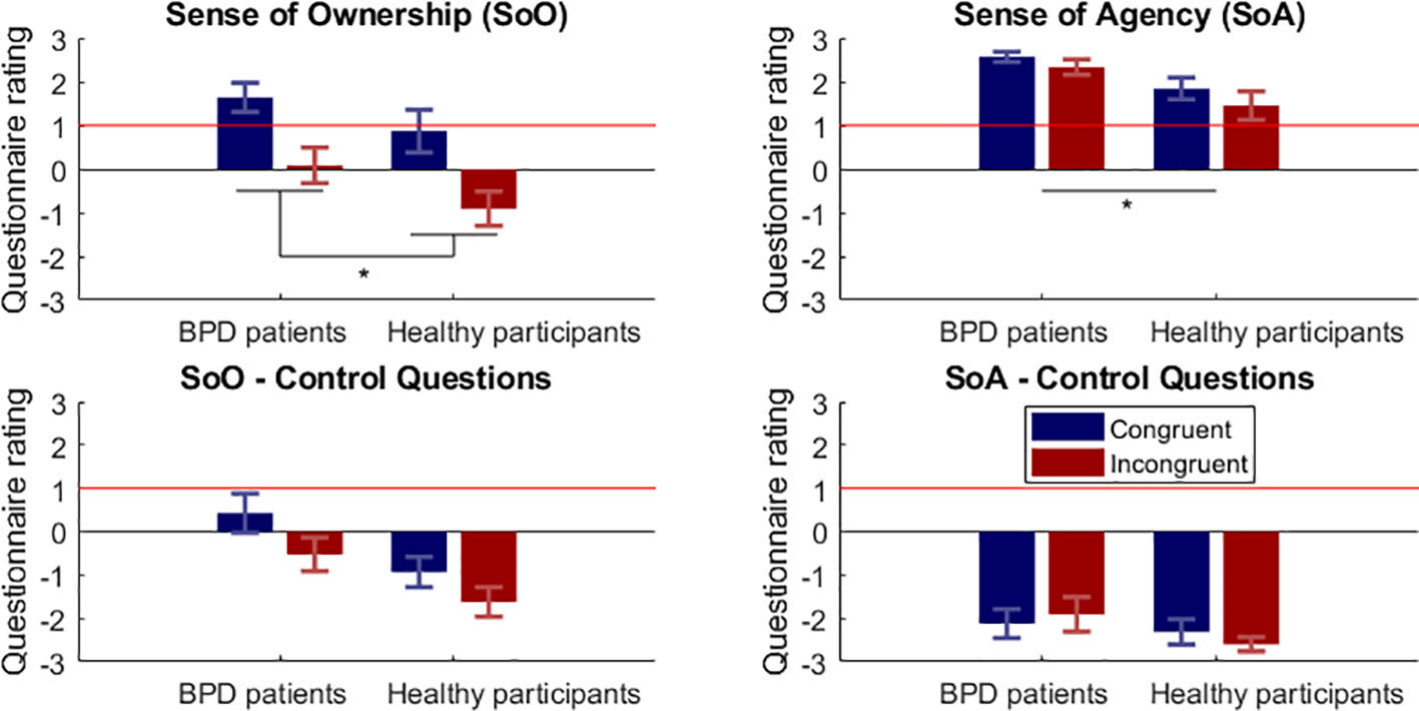
Questionnaire results for Sense of Ownership (SoO) and Sense of Agency (SoA). Depicted are the mean self-report ratings ( ± SEM) for the congruent (blue bars) and incongruent (red bars) condition¸ BPD, borderline personality disorder. The horizontal red line indicates the pre-defined illusion-criterion (≥1).*p < .05.

Questionnaire data are depicted in **Figure 2** and illusion responder rates are summarized in **Table 3**. On the group level, the SoO-induction criterion (i.e., a response of “1: rather agree” or higher on the Likert scale) was reached in the congruent condition in BPD patients (M  =  1.64; SD  = 1.52), whereas it was not reached in the congruent condition in healthy participants (M  =  0.87; SD  = 2.24), the incongruent condition in BPD patients (M  = 0 .08; SD  = 1.87), and the incongruent condition in healthy participants (M  =  −0.91; SD   = 1.80). In turn, responses to the SoO control questions showed values around zero or less throughout conditions and groups. Additional planned comparisons between the reported SoO and SoO control questions were significant in both conditions and groups (**Table 4**), confirming the illusion-specificity of the experimental manipulation. A 2 × 2 mixed ANOVA revealed a main effect of Position (F_(1,40)_ = 43.32; p <.001) with perceived SoO levels being higher in the congruent than in the incongruent condition. No significant main effect of Group (F_(1,40)_ = 2.86; p = .099) and no interaction effect (F_(1,40)_ = 0.18; p = .675) were found.

**Table 3:** Percentage of participants who passed the illusion criterion.

Condition	Group	Construct	Illusion criteria fulfilled
Congruent	BPD	Sense of ownership	76.19%
Congruent	Healthy participants	Sense of ownership	71.43%
Incongruent	BPD	Sense of ownership	38.10%
Incongruent	Healthy participants	Sense of ownership	28.57%
Congruent	BPD	Sense of agency	100%
Congruent	Healthy participants	Sense of agency	76.19%
Incongruent	BPD	Sense of agency	90.48%
Incongruent	Healthy participants	Sense of agency	76.19%

BPD, borderline personality disorder.

**Table 4:** Pairwise comparisons between sense of ownership (SoO) vs. sense of agency (SoA) questions and control questions.

Comparison	Condition	BPD patients	Healthy participants
SoO vs. SoO control	Congruent	t_(20)_ = 4.12; p < .001	t_(20)_ = 3.35; p = .002
SoO vs. SoO control	Incongruent	t_(20)_ = 1.81; p = .042	t_(20)_ = 2.19; p = .020
SoA vs. SoA control	Congruent	t_(20)_ = 13.16; p < .001	t_(20)_ = 13.67; p < .001
SoA vs. SoA control	Incongruent	t_(20)_ = 8.50; p < .001	t_(20)_ = 10.75; p < .001

The SoA-induction criterion was reached in all conditions for both groups. Highest SoA was reported for the congruent condition in BPD patients (M  =  2.57; SD  = 0.53) and lowest SoA for the incongruent condition in healthy participants (M  =  1.45; SD  = 1.49). The SoA control questions yielded values around zero or less. Multiple planned comparisons between reported SoA and SoA control questions were significant in both conditions and both groups (**Table 4**), thus again confirming the illusion-specificity of the manipulation. An ANOVA revealed a main effect of Group (F_(1,40)_ = 8.87; p = .005) in that patients with BPD reported higher SoA than healthy participants. No main effect of Position (F_(1,40)_ =  3.01; p  = 0.09), and no interaction effect (F_(1,40)_  =  0.18; p  = .673) were found.

In the single-item stress questionnaire, average values varied from M = 2.10 (SD = 1.22) in the incongruent condition in healthy participants to M = 4.38 (SD = 1.47) in the congruent condition in BPD patients. An ANOVA revealed a main effect of Position (F_(1,40)_ = 14.03; p <.001) as well as a main effect of Group (F_(1,40)_ = 14.38; p <.001), but no interaction effect (F_(1,40)_ = 0.07; p = .791).

### Intentional Binding

**Figure 3:**
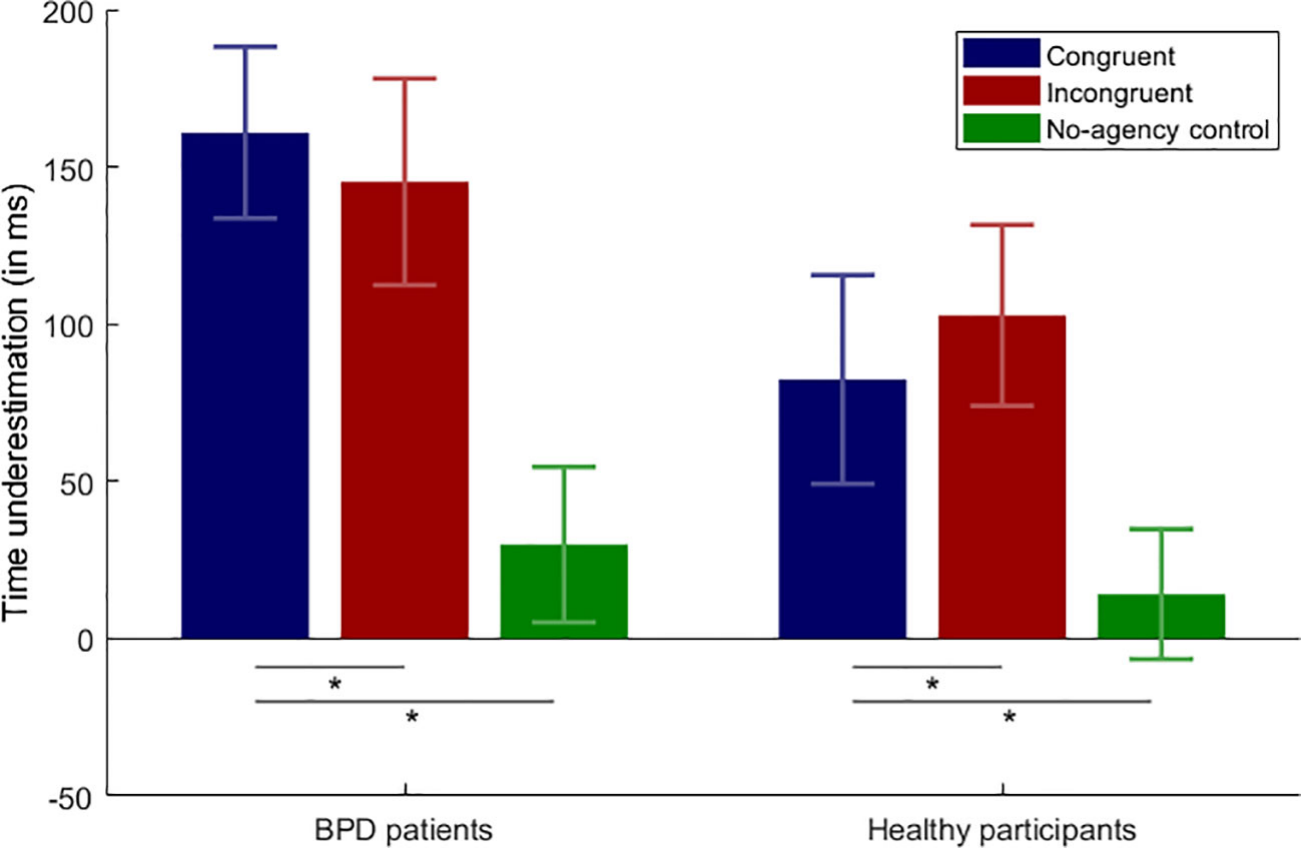
Intentional binding results. Average underestimation of time intervals in milliseconds and the respective standard error of the mean (SEM) over the experimental conditions; BPD, borderline personality disorder; *p < .05.

Intentional binding results are depicted in **Figure 3**. Time interval underestimations between the button presses and subsequently played sounds were observed in all four experimental conditions. The strongest time interval underestimation was found for the congruent condition in BPD patients (M = 160.88 ms; SD = 124.95 ms) and the weakest for the congruent condition in healthy participants (M = 82.40 ms; SD = 152.18 ms). Almost no time interval underestimation, in turn, occurred for the no-agency control condition where the time interval estimation was quite accurate and varied between M = 29.90 ms (SD = 113.26 ms) for the BPD patients and M = 14.13 ms (SD = 94.70 ms) for the healthy participants. Planned pairwise comparisons showed that in all of the four experimental conditions the time interval estimations were significantly shorter than in the no-agency control condition. Significant differences were found for the congruent condition in BPD patients vs. no-agency control condition (t_(20)_  =  5.10; p  < .001), incongruent condition in BPD patients vs. no-agency control condition (t_(20)_  =  4.08; p  < .001), congruent condition in healthy participants vs. no-agency control condition (t_(20) _ =  2.47; p  =  .011), and for the incongruent condition in healthy participants vs. no-agency control condition (t_(20)__ _=  4.11; p  =  < .001). The 2 × 2 mixed ANOVA neither revealed a main effect of Position (F_(1,40)_ = .03; p = .865), a main effect of Group (F_(1,40)_ = 2.18; p = .148), nor an interaction effect (F_(1,40)_ = 1.68; p = .203).

### EDA Analysis

**Figure 4:**
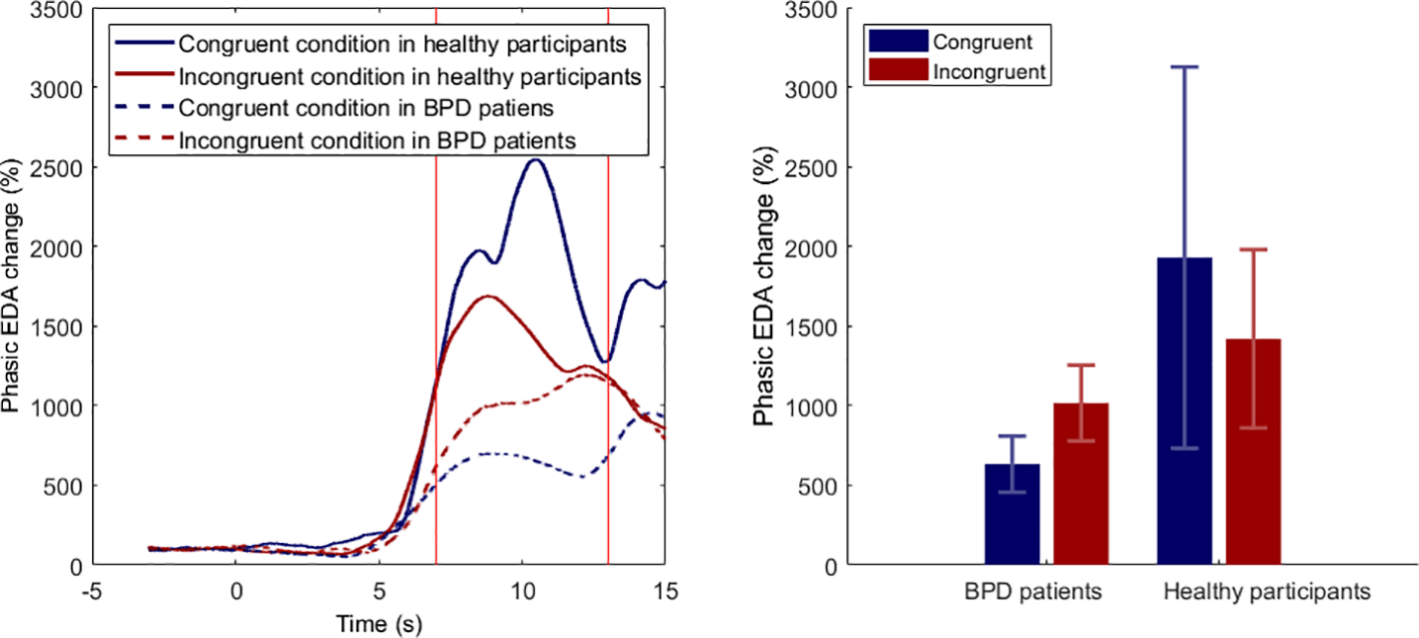
Phasic electrodermal activity (EDA) responses during syringe applications. Left panel. Phasic EDA changes, relative to a −3 to −1 baseline response. Timepoint 0 indicates beginning of the syringe application. Right panel. Mean phasic EDA changes ( ± SEM) for time interval from 7 to 13 s; BPD, borderline personality disorder.

Phasic EDA responses are shown in **Figure 4**. A phasic EDA increase shortly after the syringe application was observable in all four experimental conditions. The strongest EDA increase was observable in the congruent condition in healthy participants (M = 1929.39%; SD = 5216.79%) and the weakest in the congruent condition in BPD patients (M = 633.11%; SD = 807.18%). The 2 × 2 mixed ANOVA neither revealed a significant main effect of Position (F1,38 = 0.01; p = .923), nor a main effect of Group (F1,38 = 1.74; p = .193), nor an interaction effect (F1,38 = 0.47; p = .496).

### Associations Between Measures

Pearson correlations without Bonferroni adjustments between the different implicit and explicit SoO and SoA measures are depicted in **Table 5**. A high (r ≥ .50) positive correlation was found between SoA and SoO in the congruent condition for healthy participants (r = .85; p < .001) and in the pooled overall comparison (r = .51; p < .001) across conditions. No further significant correlations were found for any of the remaining comparisons.

**Table 5:** Pearson-correlations between sense of ownership (SoO) and sense of agency (SoA) measures.

	BPD patients		Healthy participants		Total
	Con**gruent condition**	Incongruent condition	Congruent condition	Incongruent condition	
SoA vs. SoO	r = .21 p = .353	r = .39 p = .082	r = .85 p <.001	r = .27 p = .243	r = .51 p <.001
SoA vs. Intentional Binding	r <.01 p = .998	r = .10 p = .663	r = −.37 p = .103	r <.01 p = .992	r > −.01 p = .978
EDA vs. SoO	r = .24 p = .291	r = −.06 p = .791	r = .22 p = .365	r = −.28 p = .243	r = .03 p = .773

Table includes Pearson correlation coefficients (r) and non-adjusted significance values (p). BPD, borderline personality disorder; EDA, electrodermal activity.

## Discussion

This study aimed to investigate SoO and SoA and their complex interplay in patients with BPD. An active variant of the rubber hand illusion (aRHI) was applied to 21 patients with BPD and 21 healthy participants, and their bodily self-experiences were assessed by different implicit and explicit SoO and SoA measures.

Our first research question addressed whether Bekrater-Bodmann et al.’s (12) and Neustadter et al.’s (13) questionnaire results of higher SoO in patients with BPD compared to healthy participants could be replicated. If so, we inquired whether this embodiment effect could also be physiologically detected by an implicit SoO measure applied in the current study (i.e., the skin conductance responses, relative to threatening the artificial hand) (28).

The analysis of SoO questionnaire data revealed an expectable effect of position on stronger SoO under anatomical hand congruency compared to anatomical incongruency. While these results are in line with previous studies (19, 20, 28) and demonstrate the necessity for an anatomical congruency between the artificial and participant’s real hand, no group effect, nor any interaction effects were found. Bekrater-Bodmann et al.’s (12) and Neustadter et al.’s (13) questionnaire findings of higher SoO in patients with BPD compared to healthy participants could thus not be replicated in the present study. One possible reason for this null finding could be a lack of statistical power. At least descriptively, BPD patients demonstrated higher SoO values compared to healthy participants in either condition. Also, the statistical result for the Group factor was only marginally non-significant (p = .099). Hence, it might be speculated that with a higher statistical power, we could have replicated Bekrater-Bodmann et al.’s (12) and Neustadter et al.’s (13) findings of stronger SoO in BPD patients compared to healthy participants.

The presumed lack of statistical power, in turn, could result from a too small sample size or suboptimal implementation of our aRHI apparatus. Of note, “only” 71.43% of the healthy participants reported SoO toward the artificial hand above our pre-defined illusion criterion in the congruent condition and across participants, the illusion criterion was not even reached. While comparable illusion responder rates have been reported in similar aRHI studies [75% in Kalckert and Ehrsson (20) and 63% in Kalckert and Ehrsson (41)], the present aRHI-responder rate was lower as in our former aRHI study, where it amounted to ~83% for a comparable condition (19). A potential reason for this lowered RHI-responder rate might be that, in the present study, the vertical distance between the artificial hand and participant’s real hand was 2 cm larger, due to a new table plate. Hence, although the present vertical distance (9.5 cm) was still smaller as in comparable aRHI studies (20, 41), and although the RHI vividness for the healthy participants was quite comparable, if not higher, to that reached in Bekrater-Bodmann et al.’s (12) and Neustadter et al.’s (13) studies, statistical power might have been lost by this change. As known from the literature, there is a “spatial distance rule” according to which a larger vertical distance between the artificial hand and participant’s real hand leads to lower RHI-responder rates (40). Moreover, it might be speculated that the medium size of the artificial hand used, was perhaps not optimally suitable for all our participants. For these reasons, it might be speculated that statistical power was lost due to a suboptimal aRHI apparatus.

Similar to the SoO questionnaire, the analysis of EDA data also revealed a null finding. Also for this implicit SoO measure, we could not find any group differences. In particular, we could not find any evidence for stronger fear responses relative to our syringe applications in patients with BPD compared to healthy participants. This could either point to a rather low level of artificial hand embodiment (in line with the SoO findings), or be due to methodological issues. While we consider the syringe application test a valid procedure in most RHI settings, it was perhaps less suitable in the present study. A potentially confounding factor could be that many BPD patients not only show self-injury behavior and reduced pain perception (48), but sometimes, they even experience threats as being moderating for their inner tension levels (49). Hence, it might be speculated that our BPD patients were actually not that afraid of our syringe applications which potentially also resulted in lower phasic EDA responses. For future studies, it would be interesting to investigate how the EDA response would change if the artificial hand was just gently touched instead of being pricked by the experimenter.

The second research question addressed whether besides SoO, also SoA was altered in patients with BPD, given the strong interplay and commonalities between both measures. Regarding SoA questionnaire data, statistical analysis indeed revealed a group effect for elevated SoA ratings in BPD patients compared to healthy participants. On average, patients with BPD reported higher SoA levels compared to healthy participants, regardless of whether the artificial hand was anatomically aligned, or not. On the phenomenological level, SoA informs an agent about their causal influence onto the world, but this information is, of course, a fallible representation of objective reality (7, 50). Given the present finding, one could conclude that BPD patients tend to overestimate their agentive contributions to the world, in other words, show a stronger discrepancy between their experienced levels of agency and their actual levels of agency. This conclusion, however, contradicts with the only existing theoretical account on SoA in BPD that argues for reduced SoA in patients with BPD (51). According to this account, SoA *“is often disrupted in borderline personality by a pattern in which impulses are acted upon so immediately that the self is not experienced as the author of the act”* (51). That is, in face of their strong affects, so the argument, patients with BPD are unable *“to make sense of or explain their behaviors”* (p. 937). In attempting to follow this line of reasoning, but concomitantly explaining our present SoA results, we may perhaps object that the just described form of SoA loss in BPD only partly contradicts our finding, since our experiment surely did not represent an emotional extreme situation for our BPD patients. This objection, however, only explains why we did not find reduced SoA in our experiment, whereas it lacks reason for why we find elevated SoA in our BPD patients. A potential explanation for elevated SoA could perhaps be that although SoO and SoA may dissociate in some situations (19, 20), they typically co-occur, respectively, overlap in experience and often strengthen each other (7). Hence, it may be speculated that the present SoA finding is perhaps just a side effect of a (although non-significant) SoO increase.

If SoA is elevated in BPD patients, this effect should also be implicitly demonstrable in our intentional binding measure. This was, however, not the case. Although the time intervals were clearly underestimated in all experimental conditions, which clearly indicate the expected subjective compression of time (36), no significant group effect, nor any interaction effect were found. Several reasons might account for the discrepancy between the two SoA measures. First, there might be methodological issues with the intentional binding measure. Although most studies consider intentional binding a reliable and valid SoA measure (36, 52, 53), other studies have reported temporal binding effects in the absence of voluntary actions (54, 55) or only found trend effects when attempting to replicate the presumed intentional binding mechanism (19, 34). To explain this variability in results, some authors like Buehner (54) have therefore proposed that in fact causal inference rather than agentive inference accounts for most of the observed temporal binding effect. That is, temporal binding does not necessarily require self-induced actions, but may result from any assumed causal relationship between two sensory events. Hence, if Buehner’s (54) alternative temporal binding explanation holds true, the intentional binding paradigm is perhaps a less exclusive SoA measure, as widely presumed.

Another argument for the observed discrepancy between our SoA questionnaire result and our intentional binding null finding might be that whereas intentional binding was directly assessed after every button press trial, our questionnaire data were acquired after every block, and thus more heavily relied on a postdictive SoA evaluation. To acquire self-report data more directly, an additional quick online questionnaire during the block that allows collecting data during the immediate RHI experience should therefore be considered for future studies. Moreover, whereas our SoA items focused more on the motoric experience of agency, intentional binding is presumed to be induced by temporal contiguity and temporal predictability (36). As pointed out in a previous study (19), this mismatch and difference of operationalization makes both measures only partly comparable.

In sum, the present study provides self-report evidence, but no intentional binding evidence for higher SoA in BPD patients. Moreover, in contrast to Bekrater-Bodmann et al.’s (12) and Neustadter et al.’s (13) findings, our study neither reveals self-report nor electrodermal evidence for higher SoO in BPD patients. This inconsistency in results makes it difficult to resolve our findings with the literature. Perhaps, one explanation might be that the observed effects are driven by some generally elevated perceptual suggestibility of patients with BPD (56). That is, that BPD individuals are just generally more susceptible to perceptual illusions than healthy persons. If this is the case, Bekrater-Bodmann et al.’s (12), Neustadter et al.’s (13) and our findings represent no specific effects of an altered bodily or agentive self-awareness in BPD patients, but instead some more generic illusion-susceptibility of patients with BPD. Another related explanation might be that BPD patients show a stronger response bias in their self-reporting behavior, for instance, an acquiescence bias or an extreme response bias. This assumption would not only be consistent with many aspects of the BPD psychopathology (e.g., black and white thinking, excessive emotionality), but there is also some empirical indication for a response bias in the present and previous studies (12, 13): In all three studies, BPD patients showed descriptively weaker negations for the SoO control questions than healthy control participants. Hence, although in Neustadter et al.’s (13) study, the effect of stronger SoO in BPD patients persisted after correcting for this response bias, it appears that the high SoO affirmations found in BPD patients are partly due to an acquiescence or extreme response bias.

Finally, we explored whether the explicit and implicit measures of SoO and SoA align in patients with BPD as well as in healthy participants. One finding was that across conditions, there was a moderate correlation between SoO and SoA. This finding is in line with previous studies (19, 20, 28, 39) and suggests that both measures do not relate to distinct, but to rather overlapping aspects of phenomenal experience [for a critical discussion, see Braun et al. (28)]. On a descriptive level, the correlation between SoO and SoA for the congruent condition thereby appeared to be much lower in the BPD patients (r = .21) than healthy participants (r = .85). While the most straightforward explanation for this lowered SoO/SoA correlation in the BPD patients could perhaps be a ceiling effect of the SoA values, an alternative explanation, could, however, also be a complex interplay between SoO and SoA in BPD which warrants further investigation.

## Conclusions

To our knowledge, this is the first study that specifically investigated SoO and SoA in patients with BPD, using an aRHI paradigm. While the present results show some indication for an elevated SoA in patients with BPD, no significant effects for elevated SoO could be found, which is inconsistent with the literature. Given the present data, the results are best explainable by an elevated perceptual suggestibility of patients with BPD.

## Data Availability Statement 

The raw data will be made available by the authors, without undue reservation, to any qualified researcher.

## Ethics Statement

The studies involving human participants were reviewed and approved by Medical ethics committee of the University of Oldenburg. The patients/participants provided their written informed consent to participate in this study.

## Author Contributions 

NB and TM conceptualized and designed the study. TM acquired and analyzed the data under the supervision of NB, CH, and AP. TM and NB wrote major parts of the manuscript. A-KT contributed sections to the manuscript. All authors contributed to manuscript revision, read and approved the submitted version.

## Funding

The study was conducted within a master thesis project and funded by budgets from the University of Oldenburg.

## Conflict of Interest

The authors declare that the research was conducted in the absence of any commercial or financial relationships that could be construed as a potential conflict of interest.
